# Anaplastic Lymphoma Kinase Tyrosine Kinase Inhibitor-Associated Cardiotoxicity: A Recent Five-Year Pharmacovigilance Study

**DOI:** 10.3389/fphar.2022.858279

**Published:** 2022-03-17

**Authors:** Yihan Liu, Chen Chen, Chencheng Rong, Xucheng He, Li Chen

**Affiliations:** ^1^ Department of Pharmacy, West China Second Hospital, Sichuan University, Chengdu, China; ^2^ Evidence-Based Pharmacy Center, West China Second Hospital, Sichuan University, Chengdu, China; ^3^ Key Laboratory of Birth Defects and Related Diseases of Women and Children (Sichuan University), Ministry of Education, Chengdu, China; ^4^ Department of Pharmacy, West China Hospital, Sichuan University, Chengdu, China; ^5^ West China School of Pharmacy, Sichuan University, Chengdu, China; ^6^ Department of Pharmacy, Pengzhou Second People’s Hospital, Chengdu, China

**Keywords:** anaplastic lymphoma kinase tyrosine kinase inhibitors, cardiotoxicity, FAERS, real-world study, disproportionality analysis

## Abstract

**Background:** Clinical trials frequently reported anaplastic lymphoma kinase tyrosine kinase inhibitors (ALK-TKIs) associated with cardiac adverse drug events (AEs) but minimal postmarketing data. We aimed to research real-world cardiac disorders associated with ALK-TKIs based on the Food and Drug Administration Adverse Event Reporting System (FAERS).

**Methods:** Extract reports from the FAERS from the first quarter of 2016 to the second quarter of 2021 were obtained. Data mining of cardiac disorders associated with ALK-TKIs was carried out using disproportionality analysis to determine the clinical characteristics of AEs.

**Results:** In total, 605 cases were screened out. These events were found to be more prevalent in patients ≥45 years (50.74%) and women (50.74%). The onset time of cardiac disorders was variable and concentrated within 2 months, with a median time of 33 days. The outcomes tended to be poor, with 20.93% fatality proportion. Cardiac arrhythmia was a common adverse event of ALK-TKIs, especially bradycardia. Crizotinib and lorlatinib showed positive signals in cardiac disorders, especially in heart failure, and brigatinib presented no signals. The study also found that myocarditis caused by ceritinib and cardiomyopathy caused by lorlatinib may be potential new adverse drug reactions.

**Conclusion:** ALK-TKIs were reported more frequently in cardiotoxicity than other drugs and could often manifest earlier. We also found potential new AE signals in specific drugs and need more clinical studies to confirm. Our study helps fill the safety information of ALK-TKIs in the heart and provides directions for further research.

## Introduction

Anaplastic lymphoma kinase (ALK) is a highly conserved receptor protein tyrosine kinase (RPTK) that belongs to the insulin receptor (IR) superfamily (Du et al., 2018). Most of the time, the ALK gene remains dormant, but it could cause cancers by genetic rearrangement, gene fusion, or gene overexpression once it is activated (Hallberg and Palmer, 2013). Lung cancer is the leading cause of cancer-associated deaths worldwide, and non-small-cell lung cancer (NSCLC) accounts for approximately 85% of lung cancer patients (Sung et al., 2021). Many scholars in this field found that most patients have alterations in certain driver genes, which could encode specific proteins to accelerate the proliferation of tumors. The primary ALK mutant gene in NSCLC was the echinoderm microtubule-associated protein-like 4 (EML4)-ALK fusion gene, which was found by Soda et al. (2007) and could encode EML4-ALK fusion proteins. These proteins could correspond with convoluted signals by activating multiple downstream pathways, such as PLC-γ, RAS, mTOR, and MAPK, driving aberrant proliferation and survival (Itchins et al., 2017; Lin et al., 2017). This has realized an excellent extensive perspective in NSCLC. After many years of research, ALK-TKIs have been used to treat NSCLC patients with the ALK mutant gene, which could inhibit the growth of tumor cells by inhibiting tyrosine kinase and thereby destroying the signaling pathway (Lin et al., 2017). Further research into ALK-TKIs revealed mutations of the ALK gene in other types of tumors, and the use of ALK inhibitors may produce good efficacy, for example, in anaplastic large-cell lymphoma (Mossé et al., 2013) and inflammatory myofibroblastic tumors (Butrynski et al., 2010).

Over the past dozen years, anaplastic lymphoma kinase tyrosine kinase inhibitors (ALK-TKIs) have been developed and have achieved ideal clinical outcomes via copious clinical trials (Kwak et al., 2010; Iragavarapu et al., 2015; Wu et al., 2018). Crizotinib was a multi-targeted TKI with activity against MET, ALK, and ROS1 and was the first ALK-TKI approved by the FDA for metastatic NSCLC positive for ALK rearrangements (Du et al., 2018). Because of many reasons, such as secondary mutations, tumor heterogeneity, and driving gene conversion, which may cause drug resistance (Doebele et al., 2012), second- and third-generation ALK-TKIs have been researched and developed. The U.S. Food and Drug Administration (FDA) has approved five ALK-TKIs, namely, crizotinib, ceritinib, alectinib, brigatinib, and lorlatinib. The three generations of ALK-TKIs greatly enriched the therapeutic regimens of ALK-positive cancer patients, but drug selection needs to consider many factors, among which the toxicity profile was one of the critical considerations.

With further studies of ALK-TKIs in clinical trials and increasing use in clinical practice (Costa et al., 2018; Kassem et al., 2019; Breadner et al., 2020), despite the benefits of ALK-TKIs, many adverse events (AEs) have been described, which mainly involve the gut, lung, liver, heart, nerve, and skin and have a potential effect on any other tissues (Hou et al., 2019; Wang and Wang, 2021). As an essential organ that provides sufficient blood flow to other organs and tissues, supplies oxygen and various nutrients, and maintains the normal metabolism and function of cells, the heart plays a significant role in the human body, especially in cancer patients. In several clinical trials of ALK-TKIs (Solomon et al., 2014; Camidge et al., 2018; Solomon et al., 2018; Cho et al., 2019; Zhou et al., 2019), cardiotoxicity associated with these drugs concentrated on bradycardia and QT prolongation, with an incidence of approximately 9–15%, and the severity was usually less than grade 2, with a probability of grades 3 to 4 of roughly 1–4% (Costa et al., 2018).

In clinical trials of ALK inhibitors, the cardiotoxicity associated with these drugs had mainly focused on bradycardia and QT prolongation, with a low incidence and severity usually less than grade 2. However, due to the small sample size and short observation time, clinical trials were often unable to reveal the complete safety information of drugs. Within a few years after the marketing of these drugs, several pieces of literature in clinical practice showed that cardiotoxicity associated with ALK-TKIs had a more comprehensive range, with different incidences and severities, and sometimes it could even lead to severe heart failure (Fukuizumi et al., 2015).

With the increasing variety and indication of ALK-TKIs, it was necessary to update the understanding promptly and provide an overview of the risk and characteristics of cardiac AEs for further prevention and management. However, most evidence of ALK-TKI-associated cardiotoxicity stemmed from case reports and clinical trials. We mainly needed real-world data to complement or verify clinical trial information and fully assess the cardiac safety of these drugs.

The FDA Adverse Event Reporting System (FAERS), a postmarketing database, is used to store AE reports associated with FDA-approved therapy reported spontaneously in the real world by healthcare professionals or patients and lawyers. The pharmacovigilance system of FAERS was critical for continuous monitoring of the relationship between AEs and drugs (Kamitaki et al., 2021). Therefore, this study was based on the FAERS database. We conducted disproportionality analysis (van Puijenbroek et al., 2002) to dissect the cardiac AE signals submitted to the FAERS to identify potential cardiotoxicities and assess the correlations between different ALK-TKIs and cardiac AEs. From the perspective of the real world, this study’s purpose was to assess ALK-TKIs in a more realistic environment.

## Materials and Methods

### Data Source

The data of this retrospective pharmacovigilance study were retrieved from the FAERS, and the time horizon was from the first quarter of 2016 to the second quarter of 2021. The reporters could be healthcare providers (physician, pharmacist, health professional), and non-healthcare providers (lawyer, consumer). Much helpful data files were offered to us: report source (PRSR), demographic and administrative information (DEMO), drug information (DRUG), preferred terms (PTs) coded for the adverse event (REAC), therapy start dates, and end dates for reported drugs (THER), patient outcomes (OUTC), and indications for drug administration (INDI).

According to the recommendation of the FDA, when the PRIMARYID (the number for identifying a FAERS case) was the same as the FDA_DT (date FDA received case) in the FAERS data files, we removed the datum that occurred repeatedly and selected the latest one. Then, we obtained 7,621,815 cases that used the keywords associated with the target drugs. The keywords included generic names and brand names of the ALK-TKIs, which were registered in the FDA. Through ROLE_COD (code for drug’s reported role event) in DRUG files, we chose the cases that the target drug that was considered the primary suspected drug (PS). In addition, to prevent the situation in which irregular and diverse terms of AEs would interfere with our study, we transformed the submitted AE names to the standard preferred terms (PT) using the Medical Dictionary for Regulatory Activities (MedDRA). Finally, 29,964 cases were retained for subsequent research. MedDRA is a standard set of terms covering a wide range of AE terms and is the most widely used AE coding dictionary in international studies. MedDRA was attributed and associated through five layers, from top to bottom: system organ class (SOC), high-level group term (HLGT), high-level term (HLT), preferred term (PT), and lowest level term (LLT). The higher the level, the smaller the number of terms and the wider the range. To reduce confusion and deviation and better assess the association between ALK-TKIs and cardiotoxicity, our study was mainly based on cardiac disorder SOC and the level of HLGT contained in it, and all PTs were also included in the study.

### Definition of Cardiac Adverse Events

Based on MedDRA, the SOC we researched was “cardiac disorders (10007541).” The associated HLGT was as follows: “heart failures (10019280),” “coronary artery disorders (10011082),” “pericardial disorders (10034468),” “myocardial disorders (10028593),” “cardiac arrhythmias (10007521),” “cardiac valve disorders (10046973),” “cardiac disorders, signs, and symptoms NEC (10082206).” The associated PT was as follows: “cardiac failure acute [10007556),” “cardiopulmonary failure (10051093),” “left ventricular failure (10024119),” “pericardial effusion (10034474),” “cardiac tamponade (10007610),” “pericarditis (10034484),” “cardiomyopathy (10007636),” “bradycardia (10006093),” “sinus node dysfunction (10075889),” and “sinus bradycardia (10040741).”

### Data Mining

On account of the principle of disproportionality analysis, we used the reporting odds ratio (ROR) method (Bate and Evans, 2009) to compare the whole drug-associated AEs with the reported AEs of the target drug and intend to recognize the valid connections between the investigated drugs and the reported AEs. We also performed a sensitivity analysis, which only included cases from healthcare professionals. It was regarded as a detected signal only if an AE met the algorithm’s criteria before and after sensitivity analysis simultaneously.

### Statistical Analysis

Descriptive analyses were utilized to summarize the clinical characteristics of the patients with ALK-TKI-associated cardiac AEs collected from the FAERS database, and the continuous variable was represented by mean ± standard deviation (SD). The nonparametric test (Kruskal–Wallis test) was used to compare the onset time of ALK-TKI-associated cardiac AEs and the contingency table Chi-square test or Fisher’s test was used to assess whether the fatality proportions of patients with ALK-TKI-associated cardiac AEs were different. All statistical analyses were conducted by IBM^®^ SPSS^®^ Statistics (version 26). Differences with *p* values <0.05 were considered statistically significant.

## Results

### Descriptive Analysis

The FAERS database has recorded 7,621,815 cases from the first quarter of 2016 to the second quarter of 2021, including 605 cases of cardiac disorders associated with ALK-TKIs. We summarize the characteristics of patients in these 605 cases, which are summarized in the form of charts (Table 1). The chart shows little difference in patient characteristics between each ALK-TKI. Most of the cases came from North America (32.07%), Asia (33.39%), and Europe (28.93%) and were mainly reported by healthcare professionals (78.35%). The patients affected were more often female (50.74%) than male (39.67%), with 9.59% of the events gender unknown or missing. Moreover, except for 23.47% of the age data not available, the age of patients with cardiac disorders remained consistent across all drugs, which tended to be approximately 45–74 years (50.74%), with an average of 62.96 ± 14.48 years.

**TABLE 1:** Characteristics in ALK-TKI-associated cardiac disorders cases^a^.

Characteristic	Cases, N (%)					
	Total	Crizotinib	Alectinib	Ceritinib	Brigatinib	Lorlatinib
Sex of patient						
Female	307 (50.74%)	134 (49.08%)	86 (53.75%)	33 (51.56%)	15 (45.45%)	39 (52.00%)
Male	240 (39.67%)	107 (39.19%)	64 (40.00%)	27 (42.19%)	15 (45.45%)	27 (36.00%)
Unknown	58 (9.59%)	32 (11.72%)	10 (6.25%)	4 (6.25%)	3 (9.09%)	9 (12.00%)
Age group (years)						
<18	2 (0.33%)	2 (0.73%)	0 (0.00%)	0 (0.00%)	0 (0.00%)	0 (0.00%)
18–44	47 (7.77%)	14 (5.13%)	12 (7.50%)	10 (15.63%)	4 (12.12%)	7 (9.33%)
45–64	182 (30.08%)	83 (30.40%)	43 (26.88%)	26 (40.63%)	6 (18.18%)	24 (32.00%)
65–74	125 (20.66%)	60 (21.98%)	25 (15.63%)	12 (18.75%)	9 (27.27%)	19 (25.33%)
>74	107 (17.69%)	61 (22.34%)	30 (18.75%)	2 (3.13%)	3 (9.09%)	11 (14.67%)
Unknown	142 (23.47%)	53 (19.41%)	50 (31.25%)	14 (21.88%)	11 (33.33%)	14 (18.67%)
Age (years)						
Mean ± SD	62.96 ± 14.48	64.92 ± 14.31	63.46 ± 14.81	56.20 ± 12.53	60.27 ± 14.51	61.51 ± 14.48
Reporters						
Healthcare professionals	474 (78.35%)	224 (82.05%)	122 (76.25%)	46 (71.88%)	21 (63.64%)	61 (81.33%)
Non-healthcare professionals	120 (19.83%)	46 (16.85%)	38 (23.75%)	10 (15.63%)	12 (36.36%)	14 (18.67%)
Unknown	11 (1.82%)	3 (1.10%)	0 (0.00%)	0 (0.00%)	0 (0.00%)	0 (0.00%)
Reporting region						
Asia	202 (33.39%)	83 (30.40%)	56 (35.00%)	28 (43.75%)	1 (3.03%)	34 (45.33%)
North America	194 (32.07%)	87 (31.87%)	61 (38.13%)	7 (10.94%)	15 (45.45%)	24 (32.00%)
Europe	175 (28.93%)	89 (32.60%)	39 (24.38%)	19 (29.69%)	13 (39.39%)	15 (20.00%)
South America	14 (2.31%)	10 (3.66%)	1 (0.63%)	2 (3.13%)	1 (3.03%)	0 (0.00%)
Oceania	9 (1.49%)	2 (0.73%)	1 (0.63%)	1 (1.56%)	3 (9.09%)	2 (2.67%)
Africa	2 (0.33%)	1 (0.37%)	1 (0.63%)	0 (0.00%)	0 (0.00%)	0 (0.00%)
Unspecified	9 (1.49%)	1 (0.37%)	1 (0.63%)	7 (10.94%)	0 (0.00%)	0 (0.00%)

a N: the number of cases; SD: standard deviation.

### Signal Detection

The signal detection of ALK-TKIs associated with cardiac disorders (SOCs) is shown in Table 2. According to the aforementioned criteria for signal occurrence, both crizotinib and lorlatinib generated signals before and after sensitivity analysis. We summarized the HLGT of these drugs involved in cardiac disorders, and the calculation results of the disproportionality analysis are listed in Table 3. The four kinds of HLGT presented a significant association with ALK-TKIs for heart failures, pericardial disorders, myocardial disorders, and cardiac arrhythmias. From the total data of all ALK-TKIs, only heart failure and pericardial disorders had positive signals. In terms of individual ALK-TKIs, the drugs associated with heart failure were lorlatinib and crizotinib, of which lorlatinib had a stronger association than crizotinib. Except for brigatinib, the other four drugs could cause pericardial disease, of which ceritinib appeared to have the strongest relevance, and alectinib was the last. In addition, the drugs involved in cardiac arrhythmias include crizotinib and alectinib, among which crizotinib had a stronger relevance. We should also note that only lorlatinib could cause myocardial disorders.

**TABLE 2:** Signal detection of cardiac disorders^a^.

Regimen	^b^Without sensitive analysis		^c^With sensitive analysis	
	N	ROR (95% CI)	N	ROR (95% CI)
ALK-TKIs	605	1.14 (1.05, 1.24)	475	1.75 (1.60, 1.92)
Crizotinib	273	1.24 (1.10, 1.40)	224	2.89 (2.53, 3.31)
Alectinib	160	1.12 (0.95, 1.31)	122	3.08 (2.56, 3.71)
Ceritinib	64	1.10 (0.86, 1.42)	47	2.66 (1.98, 3.58)
Brigatinib	33	0.63 (0.44, 0.89)	21	1.41 (0.91, 2.17)
Lorlatinib	75	1.34 (1.06, 1.70)	61	2.97 (2.29, 3.86)

a *CI*: confidence interval; *N*: the number of cases; *ROR*: reporting odds ratio.

b AE reporters were not limited, including healthcare providers and non-healthcare providers.

c AE reporters were limited in healthcare providers.

**TABLE 3:** Signal detection of HLGT for ALK-TKI-associated cardiac disorders^a^.

Regimen	HLGT	Heart failures	Coronary artery disorders	Pericardial disorders	Myocardial disorders	Cardiac arrhythmias	Cardiac valve disorders	Cardiac disorders, signs, and symptoms NEC
ALK-TKIs	N	137	67	125	28	267	3	50
	ROR (95% CI)	1.64 (1.38, 1.94)	0.65 (0.51, 0.82)	6.66 (5.58, 7.95)	0.80 (0.55, 1.16)	1.13 (1.00, 1.28)	0.21 (0.07, 0.67)	0.41 (0.31, 0.55)
Crizotinib	N	72	28	48	8	126	2	22
	ROR (95% CI)	2.07 (1.64, 2.61)	0.65 (0.45, 0.94)	6.09 (4.58, 8.10)	0.55 (0.27, 1.10)	1.28 (1.08, 1.53)	0.34 (0.09, 1.37)	0.44 (0.29, 0.67)
Alectinib	N	31	15	30	6	83	1	9
	ROR (95% CI)	1.37 (0.96, 1.95)	0.54 (0.32, 0.89)	5.87 (4.09, 8.41)	0.64 (0.29, 1.42)	1.31 (1.05, 1.63)	0.26 (0.04, 1.88)	0.28 (0.14, 0.53)
Ceritinib	N	5	8	29	1	24	0	6
	ROR (95% CI)	0.54 (0.22, 1.30)	0.71 (0.35, 1.41)	14.19 (9.81, 20.51)	0.26 (0.04, 1.86)	0.92 (0.62, 1.38)	-	0.45 (0.20, 1.01)
Brigatinib	N	5	7	3	2	16	0	4
	ROR (95% CI)	0.61 (0.25, 1.48)	0.7 (0.33, 1.47)	1.63 (0.52, 5.05)	0.59 (0.15, 2.38)	0.70 (0.42, 1.14)	-	0.34 (0.13, 0.92)
Lorlatinib	N	24	9	15	11	18	0	9
	ROR (95% CI)	2.72 (1.81, 4.07)	0.82 (0.42, 1.58)	7.47 (4.49, 12.42)	2.99 (1.65, 5.41)	0.71 (0.45, 1.13)	-	0.7 (0.37, 1.36)

a CI: confidence interval; N: the number of cases, ROR: reporting odds ratio.

We also selected PT, which had positive signals in the aforementioned HLGT, and the calculation results of the disproportionality analysis are listed in Table 4. Judging from the 11 types of PT, pericardial effusion occurred in all four ALK-TKIs, and the correlation of these AEs was stronger than that of other types of PT associated with cardiac AEs according to ROR ranking. There were many PT types involved in ceritinib, including pericardial effusion, cardiac tamponade, and pericarditis, and the strong ROR signals indicated that it might cause a variety of pericardial disorders. The relevance of pericarditis was the strongest, which suggested that it may be a specific AE of ceritinib. We also found that in terms of cardiac arrhythmias, ALK-TKIs mainly caused bradycardia-associated AEs.

**TABLE 4:** Positive signals of PT for ALK-TKI-associated cardiac disorders^a^.

HLGT/PT	Crizotinib		Alectinib		Ceritinib		Lorlatinib	
	N	ROR (95% CI)	N	ROR (95% CI)	N	ROR (95% CI)	N	ROR (95% CI)
Heart failures								
Cardiac failure	52	3.08 (2.33, 4.05)	-	-	-	-	15	3.40 (2.04, 5.81)
Cardiac failure acute	4	2.78 (1.04, 7.43)	-	-	-	-	-	-
Cardiopulmonary failure	3	4.38 (1.41, 13.60)	-	-	-	-	-	-
Left ventricular failure	-	-	3	6.66 (2.14, 20.70)	-	-	-	-
Pericardial disorders								
Pericardial effusion	41	9.06 (6.66, 12.34)	23	7.83 (5.19, 11.81)	19	16.06 (10.20, 25.29)	13	10.83 (6.26, 19.47)
Cardiac tamponade	-	-	5	7.41 (3.08, 17.84)	6	22.00 (9.85, 49.14)	3	10.78 (3.47, 35.11)
Pericarditis	-	-	-	-	15	26.49 (15.90, 44.12)	-	-
Myocardial disorders								
Cardiomyopathy	-	-	-	-	-	-	3	4.39 (1.41, 13.95)
Cardiac arrhythmias								
Bradycardia	56	4.89 (3.75, 6.36)	42	5.67 (4.18, 7.69)	-	-	-	-
Sinus node dysfunction	-	-	4	11.39 (4.26, 30.43)	-	-	-	-
Sinus bradycardia	12	6.38 (3.62, 11.26)	9	7.39 (3.84, 14.23)	3	6.06 (1.95, 18.82)	-	-

a CI: confidence interval; N: the number of cases, ROR: reporting odds ratio.

### Time to Onset of ALK-TKI-Associated Cardiac Adverse Effects

**FIGURE 1:**
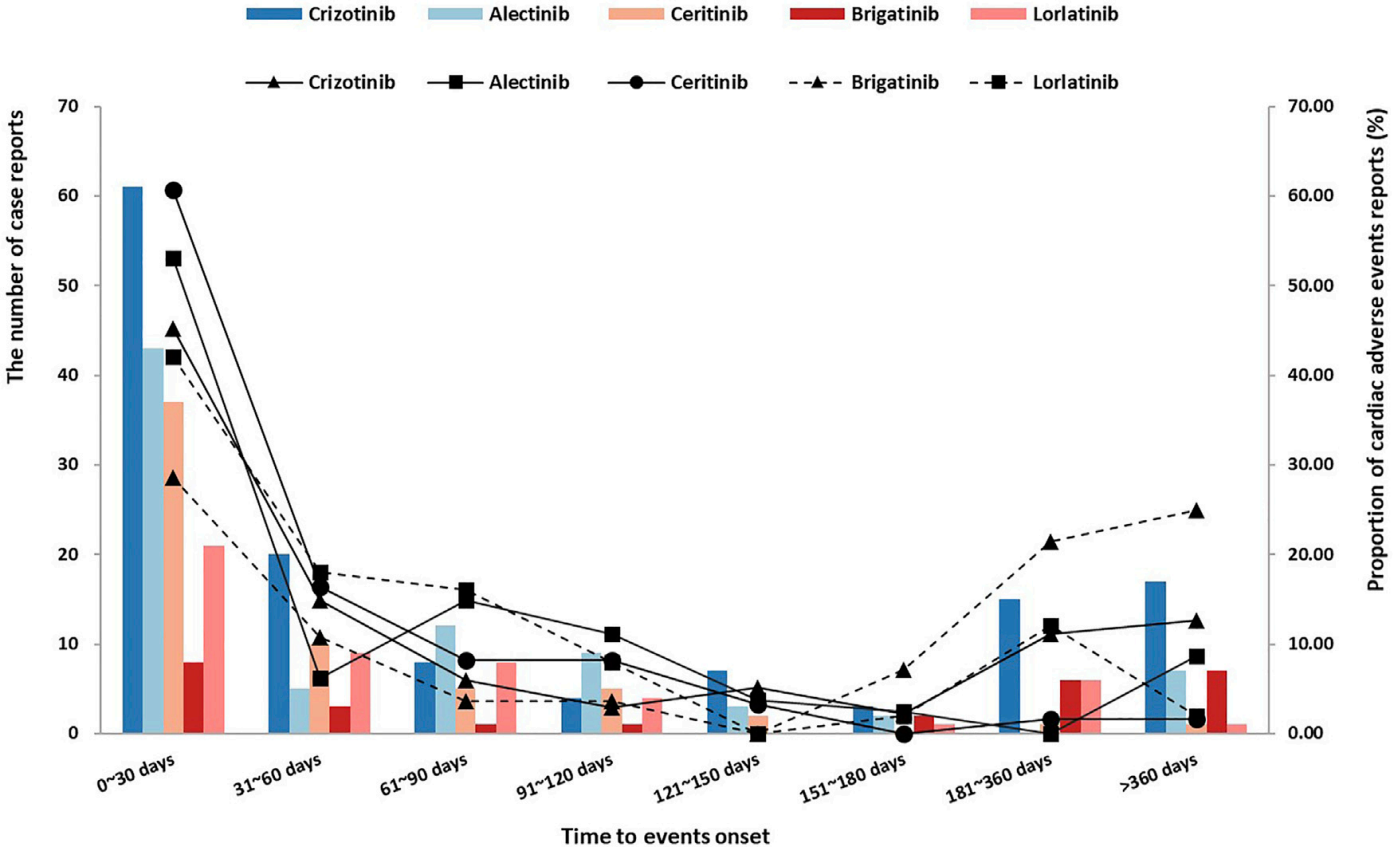
Time to ALK-TKI-associated cardiac disorder onset.

According to all ALK-TKIs, the median onset time is 33 days, and the interquartile range is 10.0–105.5 days. The distribution of onset time of all ALK-TKIs is shown in Figure 1, which was concentrated in approximately 0–30 days. Due to the difference in the time-to-market and the discrepancy in the clinical use of ALK-TKIs, only considering the number of reports may not be able to accurately compare the occurrence of AEs among the five drugs, so we not only reported the number of reports (bar chart), the proportion of onset time was also shown as a supplement (line graph). We used the Kruskal–Wallis test to describe the onset time of cardiac disorders statistically and showed a significant difference (*p* = 0.006). Interestingly, after removing the data of brigatinib, there was no significant difference in the onset time of the other ALK-TKIs (Kruskal–Wallis test, *p* = 0.083), which could be supposed that brigatinib had a longer onset time of cardiac AEs. The median (M) and interquartile range (IQR) of onset time were crizotinib (M: 40 days, IQR: 9.0–166.0 days), alectinib (M: 28 days, IQR: 10.5–96.5 days), ceritinib (M: 25 days, IQR: 7.0–60.0 days), brigatinib (M: 168 days, IQR: 19.0–365.0 days), and lorlatinib (M: 42 days, IQR: 14.0–92.25 days). Overall, 47.89% of cardiac AEs occurred within 30 days after medication, and 61.13% occurred within 60 days, but 9.30% still occurred after 365 days, so the long-term cardiotoxicity of ALK-TKIs is still worthy of our attention.

### Fatality Proportion Due To ALK-TKI-Associated Cardiac Disorders

**FIGURE 2:**
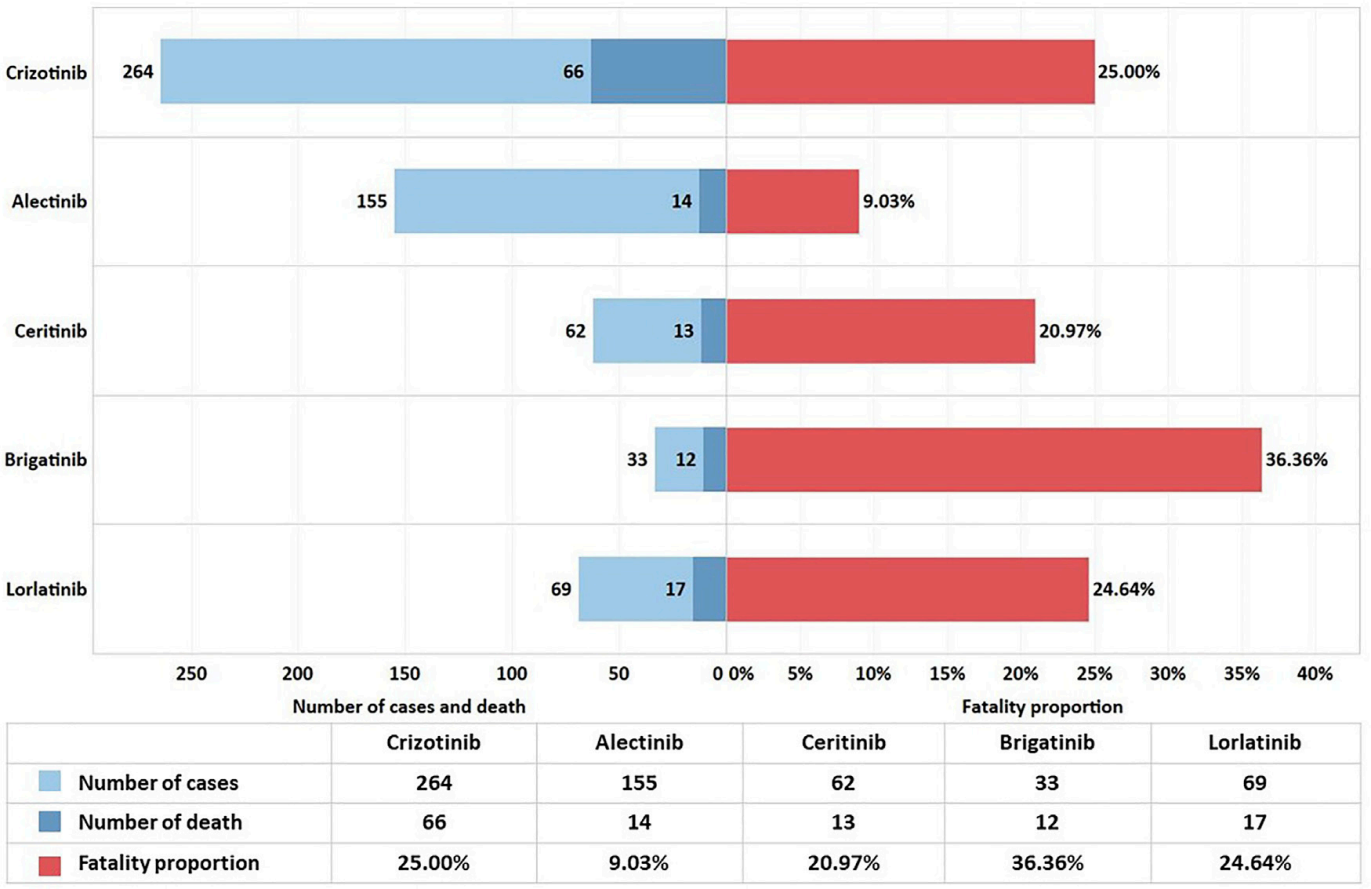
Fatality proportion from ALK-TKI-associated cardiac disorders.

To analyze the patient prognosis after cardiac AEs, we performed a statistical analysis of the outcomes recorded in the DEMO file. We chose DE (death) due to cardiac disorders regarding inpatient outcomes. We calculated the proportions of deaths and have displayed the results in Figure 2.

The study showed 20.93% fatality proportion in total, indicating that the outcome of cardiac AEs was relatively unsatisfactory. The contingency table chi-square test for frequency of death indicates a moderate correlation between different ALK-TKIs and the fatality proportion (*p* < 0.001, Cramer’s V = 0.191). The pairwise comparison results showed that the fatality proportion in the alectinib group was considerably lower than that in the other therapy groups. Except for the difference between alectinib therapy and the other four drugs, there was no significant difference between the other two groups. The fatality proportion and *p*-value between alectinib and others were as follows: alectinib and crizotinib (9.03 vs. 25.00%, *p* < 0.001), alectinib and ceritinib (9.03 vs. 20.97%, *p* = 0.016), alectinib and brigatinib (9.03 vs. 36.36%, *p* < 0.001), and alectinib and lorlatinib (9.03 vs. 24.64%, *p* = 0.002).

## Discussions

The occurrence of drug-induced cardiac disorders significantly impacts patients and is one of the leading causes of death in drug-induced diseases (Lopez-Gonzalez et al., 2009). Currently, some scholars in the international community have conducted research and assessments on the cardiotoxicity of ALK-TKIs. However, due to the limitations of clinical trials, we needed more real-world studies after marketing to improve the safety information of drugs. Therefore, this study uses disproportionality analysis to detect the ALK-TKI-associated cardiac AEs reported in the FAERS database. Among all cases of ALK-TKI-associated cardiotoxicity in this study, patients were mainly female (50.75%) and old (38.25% ≥ 65 years old, median = 65 years old). The results of data mining showed that among 5 ALK-TKIs, crizotinib and lorlatinib presented a higher risk of cardiotoxicity, with HLGT involved mainly in heart failure, pericardial disorders, myocardial disorders, and arrhythmia. Other drugs had no signal at the SOC but presented some cardiotoxicity risk at specific HLGT and PT. Ceritinib was associated with a higher risk of pericardial disorders, while alectinib was associated with a higher risk of pericardial disorders and arrhythmias. It was interesting that no signals were mined in brigatinib. We also studied the onset time and the fatality proportion, which showed ALK-TKI-associated cardiotoxicity may have the earlier time of onset and higher fatality proportion.

From the current studies, it can be perceived that old age is a risk factor for various cancers and decreases cardiac function (Shaw et al., 2011; Ou et al., 2013), but no clinical study has shown sex differences in cardiac disorders caused by ALK-TKIs (Ou et al., 2013; Ou et al., 2016). Patients aged 45–64 years (30.08%) also accounted for a high proportion, which may be associated with the high frequency that patients with NSCLC harboring the EML4-ALK fusion gene were observed in relatively younger patients (Baldi et al., 2014; Toyokawa and Seto, 2014).

According to our analysis of FAERS data, there were few types of cardiac AEs caused by these drugs, but the characteristics of cardiac AEs of the five ALK-TKIs were different. Among all ALK-TKIs, crizotinib and lorlatinib presented stronger cardiotoxic signals, and the type of cardiotoxicity involved in them was synchronous. In terms of HLGT, these two drugs had significant signals in heart failure and pericardial disorders. For serious cardiac AEs such as HLGT of heart failure (HF), fewer studies have reported HF induced by these drugs, just a few cases have been reported, and some studies have shown that there is nothing to do with the treatment of ALK-TKIs. Nevertheless, this may be associated with the physiological status of the patients. If the patient had risk factors such as older age and heart disease before therapy, it should be noted that ALK-TKIs may lead to HF in clinical practice (Fukuizumi et al., 2015). From the perspective of pericardial disorders, in addition to crizotinib and lorlatinib, alectinib and ceritinib also showed strong signals in this HLGT, and it mainly involved pericardial effusion and cardiac tamponade. Existing clinical studies have shown pericardial effusion and cardiac tamponade be life-threatening complications of NSCLC, and severe pericardial effusion was indicative of advanced cancer (Atar et al., 1999; Wang et al., 2000; Mufti et al., 2018). At present, there was one case report on pericardial effusion caused by alectinib (Ulhoi et al., 2021), but more case reports have shown that the symptoms of pericardial effusion can be effectively improved using ALK-TKIs (Gadgeel et al., 2014; Hao et al., 2015; Matsuo et al., 2016; Kasai et al., 2018). Our analysis indicated a relationship between ALK-TKIs and the aforementioned pericardial disorders, which was most likely a false-positive result caused by complications of the original disease. In addition to these common signals, crizotinib was associated with a higher risk of arrhythmia, and lorlatinib was associated with a higher frequency of myocardial disorders. Arrhythmia was a common adverse drug reaction of ALK-TKIs in recent studies, mainly including bradycardia, QT prolongation, and atrioventricular (AV) block, and most of them were assessed as less than or equal to grade 2, but rare serious AEs still possibly exist (Atar et al., 1999; Fukuizumi et al., 2015; Bauer et al., 2019; Gaillard et al., 2019). It has been reported that the decrease in the heart rate (HR) was a pharmacodynamic phenomenon of crizotinib, and every 100 ng/ml increase in plasma crizotinib concentration reduced the HR by an average of 2.5 bpm, which could be explained by the antagonist effect of the drug on L-type calcium channels, a chronotropic effect on a sinoatrial node, or an anti-mesenchymal epithelial transition effect of crizotinib (Nickens et al., 2011; Ou et al., 2013). A global, randomized, phase 3 clinical trial (Shaw et al., 2020) showed a significant difference in the incidence of bradycardia arrhythmia with crizotinib and lorlatinib (12 vs. 1%). Another ongoing phase I/II study involved 295 healthy volunteers with therapeutic of lorlatinib (ClinicalTrials.gov identifier: NCT01970865) (Bauer et al., 2019), two patients (0.7%) were reported to have asymptomatic first-degree AV block (grade 1 in severity), one patient (0.3%) was reported to have grade 3 that led to temporary discontinuation from treatment, and it should be noted that this patient had pre-existing second-degree AV block. QT prolongation was reported in 19 patients (6.4%), most of which were less than grade 2, although one event was grade 3 in severity and required temporary discontinuation. None of these events resulted in permanent treatment discontinuation. All the aforementioned studies showed a low risk of arrhythmia caused by lorlatinib, which was consistent with the results of our study. No positive signals of QT prolongation or atrioventricular block were detected in this study, and only alectinib and crizotinib produced positive signals of cardiac arrhythmia. The reason may be that QT prolongation here does not belong to cardiac disorders (SOCs), but some cardiac and vascular investigations (excluding enzyme tests) or cardiac arrhythmias caused by other drugs were milder and hard to be identified as drug-induced AEs. Lorlatinib-caused cardiomyopathy is not shown in the FDA package inserts. There was no description in the literature of cardiomyopathy associated with lorlatinib, and the results of our study suggested that it was necessary to verify it through further standard tests.

Although no signal was detected at SOC for the other three drugs, except for brigatinib, alectinib and ceritinib had certain signals at specific HLGT and PT. It is worth noting that the signals of ceritinib-caused pericarditis were also not included in the FDA package inserts. Remarkably, the high ROR value of pericarditis presents a strong correlation with ceritinib, suggesting that this might be a specific adverse drug reaction of ceritinib. A case report reported that after 2 months of ceritinib, a female patient developed acute respiratory distress with pericarditis and pleurisy, resulting in delayed hypersensitivity reaction, which was confirmed to be an adverse drug reaction caused by ceritinib (Gaillard et al., 2019). The result of our study and fewer case reports suggested that pericarditis may be a potentially rare, specific adverse drug reaction to ceritinib, and more studies are needed to confirm the association.

The onset time of these drugs was variable. Except for brigatinib, there was no significant difference in the distribution of onset time of the other four drugs, and it was mainly within 2 months after the medication, with a median time of 33 days. It was suggested that cardiac AEs might occur shortly after the first administration of these drugs. The median time of brigatinib was 168 days which was different from others. However, few studies have examined the onset time of ALK-TKI-associated cardiotoxicity. Our study showed that brigatinib had a longer onset time of cardiotoxicity than others, so the reason why we did not detect any signals of brigatinib might be that it has long-term cardiotoxicity, and our observation was relatively short. Indeed, we need more cases and studies to confirm this. Our study also indicated that patients with cardiac AEs associated with ALK-TKIs usually had a poor prognosis, with a death proportion of 20.92%. However, it should be noted that the death proportion of alectinib (9.03%) was the lowest, which was significantly different from others, suggesting that alectinib may cause a relatively small risk of death after cardiac AEs. At present, clinical studies have also shown that alectinib therapy is well tolerated and does not prolong the QT interval or cause clinically relevant changes in cardiac function (Morcos et al., 2017). It is worth noting that the results of data mining showed that brigatinib did not detect any signal at any level, but the fatality proportion was significantly higher than other drugs. We think it might be because of its less toxicity to the heart and the AEs were not monitored during medication, resulting in underreporting, which made the number of reports in the denominator part low. In addition, according to the instructions, clinical treatment guidelines, and actual clinical medication, brigatinib is mainly used to treat ALK rearrangement in NSCLC. Although it is listed as first-line therapy in the NCCN guideline, it belongs to “other recommendations.” Clinically, brigatinib also showed significant advantages after the failure of alectinib, crizotinib, and other ineffective or poor treatments (Hochmair et al., 2019). Therefore, the cardiotoxicity of brigatinib we researched was likely the AEs occurred in the subsequent therapy. The patients themself were in the later stage of the disease course, and it was very reasonable that the patients would die due to the progression or complications of the disease itself. By this token, it was not surprising that the fatality proportion of patients treated with brigatinib was significantly higher.

Since FAERS did not report on patients’ treatment lines, we could not separate treatment lines for analysis. However, for a report, the reporters could fill in multiple drugs related to AEs and classify the drugs into primary suspect drug (PS), secondary suspect drug (SS), concomitant (C), and interacting (I), which were marked in the ROLE_COD column in the drug file. To minimize the deviation caused by the treatment line, we tried to exclude the reports involving other ALK-TKIs in the drug-related to the target AEs with the target drug as PS. For patients, the occurrence of the target AEs was associated with only one ALK-TKI. We found that such reports were only 6, and the resulting signal generation results were not different from those before culling. The signal detection is shown in Supplementary Tables S1–S3. Nevertheless, this approach did not fully address the various treatment stages of patients, which remains a limitation of our study.

We noted that a more extensive FAERS study of ALK-TKIs (Omar et al., 2021) was recently published where the authors analyzed all AEs of ALK-TKIs primarily at the SOC, but they did not report any cardiac AEs. The reason might be that the data mining methods we used were different. Data mining methods are mainly divided into frequency analysis and Bayesian analysis. We only used frequency analysis for research, and this study used a combination of the two methods. However, the sensitivity of Bayesian analysis is lower (Faich and Morris, 2012), especially for less-reported potential AEs that do not generate a signal, which may allow investigators to miss some rarer AEs of concern. From the perspective of clinical trials, compared with other common AEs, the number of reports and types of cardiotoxicity of ALK-TKIs is low, and the current literature often only repeated the cardiac AEs reported in clinical trials. Cardiotoxicity of ALK-TKIs is not well understood or concerned, but there are still severe and new cardiotoxicities noticed by case reports. From this point of view, if Bayesian analysis was used, there might still be no signal for these AEs, and we still ignored them. Therefore, we only use frequency analysis which has higher sensitivity, hoping to find possible overlooked significant cardiotoxicity and making up for the lack of current research. Moreover, besides SOC, we also conducted signal mining in HLGT and PT, which were discussed as secondary outcomes, to enable our research to be more detailed and provide readers with more possible research directions.

In general, according to the results of this signal detection, crizotinib and lorlatinib generated more and stronger signals, while brigatinib did not generate signals at any level. Compared with other ALK-TKIs, crizotinib and lorlatinib had poor cardiac safety, and brigatinib had a better safety profile.

### Limitations

Although data mining improved the drug safety content through real-world data, it still had some limitations: A) The FARES database was a spontaneous reporting system, and due to the different standardizations and habits of different reporters, omissions and misstatements were inevitable, which may impact the results of sensitivity analysis. B) The disproportionality method has high sensitivity but cannot confirm the causal relationship but only the statistical association between AEs and drugs. There may be the risk of reverse causality. For example, the signals of pericardial effusion and cardiac tamponade detected in this study are most likely caused by the disease progression. C) There have been many studies on data mining based on SOC classification, but SOC is not classified by specific diseases, so it may not be able to assess the safety risk of drugs in specific diseases. In addition, some cardiac-related PTs, such as electrocardiogram QT interval abnormal, belong to the SOC of investigations, rather than the SOC of cardiac disorders, which may prevent these PTs from being included in the study. D) The limitation of the reported outcome: there was no certainty between death and ALK-TKIs use, and death may be more related to disease progression. E) Clinical information such as the severity of the patient’s cancer and other treatment measures were lacking (Chen et al., 2021).

## Conclusion

Based on the FAERS database, we assessed the cardiotoxicity of five ALK-TKIs and their potential association with the characteristics involved in pathogenesis. ALK-TKIs were reported more frequently than other drugs in cardiotoxicity, suggesting potential cardiotoxicity risks which could often manifest earlier. In general, we should focus on ALK-TKI-associated HLGT, including heart failures, pericardial disorders, and cardiac arrhythmias. In all drugs, crizotinib and lorlatinib had a higher frequency of cardiotoxicity reporting, and brigatinib presented no signals. Myocarditis to ceritinib and cardiomyopathy to lorlatinib may be potential specific adverse drug reactions. We should pay close attention to patients with old age or poor cardiac function in clinical practice. Moreover, drug combinations for patients with high cardiotoxicity or reduced cardiac function should be avoided as much as possible.

## Data Availability

Publicly available datasets were analyzed in this study. These data can be found here: https://fis.fda.gov/extensions/FPD-QDE-FAERS/FPD-QDE-FAERS.html.
