# Supplementary material for: Anaplastic Lymphoma Kinase Tyrosine Kinase Inhibitor-Associated Cardiotoxicity: A Recent Five-Year Pharmacovigilance Study
Source: Front Pharmacol. 2022 Mar 17;13:858279. doi: 10.3389/fphar.2022.858279 (PMC8968911; doi:10.3389/fphar.2022.858279)
Supplement: Supplementary file 1 [file Table1.DOCX]

Supplementary Material

# Supplementary Tables

## Supplementary Table 1

**Supplementary Table 1** Signal detection of HLGT for ALK-TKI-associated cardiac disorders^a^.

| **Regimens** | **Signal detection** | |
| --- | --- | --- |
|  | **N** | **ROR (95%CI)** |
| **ALK-TKIs** | 605 | 1.14 (1.05, 1.24) |
| **Crizotinib** | 273 | 1.24 (1.10, 1.40) |
| **Alectinib** | 160 | 1.12 (0.95, 1.31) |
| **Ceritinib** | 64 | 1.10 (0.86, 1.42) |
| **Brigatinib** | 33 | 0.63 (0.44, 0.89) |
| **Lorlatinib** | 75 | 1.34 (1.06, 1.70) |
| *^a^In* ***Supplementary Table 1****, CI* confidence interval, *N* the number of cases, *ROR* reporting odds ratio | | |

## Supplementary Table 2

**Supplementary Table 2** Signal detection of HLGT for ALK-TKI-associated cardiac disorders^a^.

| **Regimens** | **HLGT** | **Heart failures** | **Coronary artery disorders** | **Pericardial disorders** | **Myocardial disorders** | **Cardiac arrhythmias** | **Cardiac valve disorders** | **Cardiac disorders, signs, and symptoms NEC** |
| --- | --- | --- | --- | --- | --- | --- | --- | --- |
| **ALK-TKIs** | N | 135 | 67 | 123 | 28 | 258 | 3 | 48 |
|  | ROR (95%CI) | 1.61 (1.36, 1.91) | 0.65 (0.51, 0.82) | 6.55 (5.48, 7.83) | 0.80 (0.55, 1.16) | 1.09 (0.97, 1.24) | 0.21 (0.07, 0.67) | 0.40 (0.30, 0.53) |
| **Crizotinib** | N | 71 | 28 | 47 | 8 | 124 | 2 | 21 |
|  | ROR (95%CI) | 2.04 (1.61, 2.58) | 0.65 (0.45, 0.94) | 5.96 (4.47, 7.95) | 0.55 (0.27, 1.10) | 1.26 (1.06, 1.51) | 0.34 (0.09, 1.37) | 0.42 (0.27, 0.64) |
| **Alectinib** | N | 30 | 15 | 29 | 6 | 81 | 1 | 8 |
|  | ROR (95%CI) | 1.32 (0.92, 1.89) | 0.54 (0.32, 0.89) | 5.67 (3.93, 8.18) | 0.64 (0.29, 1.42) | 1.27 (1.02, 1.59) | 0.26 (0.04, 1.88) | 0.25 (0.12, 0.49) |
| **Ceritinib** | N | 5 | 8 | 29 | 1 | 22 | 0 | 6 |
|  | ROR (95%CI) | 0.54 (0.22, 1.30) | 0.71 (0.35, 1.41) | 14.19 (9.81, 20.51) | 0.26 (0.04, 1.86) | 0.84 (0.55, 1.29) | - | 0.45 (0.20, 1.01) |
| **Brigatinib** | N | 5 | 7 | 3 | 2 | 15 | 0 | 4 |
|  | ROR (95%CI) | 0.61 (0.25, 1.48) | 0.70 (0.33, 1.47) | 1.63 (0.52, 5.05) | 0.59 (0.15, 2.38) | 0.65 (0.39, 1.08) | - | 0.34 (0.13, 0.92) |
| **Lorlatinib** | N | 24 | 9 | 15 | 11 | 16 | 0 | 9 |
|  | ROR (95%CI) | 2.71 (1.81, 4.07) | 0.82 (0.42, 1.58) | 7.46 (4.48, 12.42) | 2.99 (1.65, 5.41) | 0.63 (0.38, 1.03) | - | 0.70 (0.37, 1.36) |
| *^a^In* ***Supplementary Table 2****, CI confidence interval, N the number of cases, ROR reporting odds ratio* | | | | | | | | |

## Supplementary Table 3

**Supplementary Table 3** Positive signals of PT for ALK-TKI-associated cardiac disorders^a^.

| **HLGT/PT** | **Crizotinib** | | **Alectinib** | | **Ceritinib** | | **Lorlatinib** | |
| --- | --- | --- | --- | --- | --- | --- | --- | --- |
|  | N | ROR (95%CI) | N | ROR (95%CI) | N | ROR (95%CI) | N | ROR (95%CI) |
| **Heart failures** | | | | | | | | |
| Cardiac failure | 51 | 3.02 (2.29, 3.98) | - | - | - | - | 14 | 3.26 (1.92, 5.52) |
| Cardiac failure acute | 4 | 2.78 (1.04, 7.43) | - | - | - | - | - | - |
| Cardiopulmonary failure | 3 | 4.38 (1.41, 13.60) | - | - | - | - | - | - |
| Left ventricular failure | - | - | 3 | 6.66 (2.14, 20.70) | - | - | - | - |
| **Pericardial disorders** | | | | | | | | |
| Pericardial effusion | 40 | 8.84 (6.47, 12.08) | 22 | 7.49 (4.92, 11.39) | 19 | 16.06 (10.20, 25.29) | 12 | 10.39 (5.88, 18.36) |
| Cardiac tamponade | - | - | 5 | 7.41 (3.08, 17.84) | 6 | 22.00 (9.85, 49.14) | 3 | 10.78 (3.47, 35.11) |
| Pericarditis | - | - | - | - | 15 | 26.49 (15.90, 44.12) | - | - |
| **Myocardial disorders** | | | | | | | | |
| Cardiomyopathy | - | - | - | - | - | - | 3 | 4.39 (1.41, 13.95) |
| **Cardiac arrhythmias** | | | | | | | | |
| Bradycardia | 55 | 4.88 (3.75, 6.36) | 41 | 5.53 (4.06, 7.53) | - | - | - | - |
| Sinus node dysfunction | - | - | 4 | 11.39 (4.26, 30.43) | - | - | - | - |
| Sinus bradycardia | 12 | 6.38 (3.62, 11.26) | 9 | 7.39 (3.84, 14.23) | 3 | 6.06 (1.95, 18.82) | - | - |
| *^a^In* ***Supplementary Table 3****, CI confidence interval, N the number of cases, ROR reporting odds ratio.* | | | | | | | | |
